# Case Report: Maintaining a balance between vascular access patency and stable dissection status in a hemodialysis patient with unrepaired type A aortic dissection

**DOI:** 10.3389/fcvm.2025.1561645

**Published:** 2025-03-24

**Authors:** Qiquan Lai, Ling Chen, Xuejing Gao, Hongtao Tie, Ziming Wan

**Affiliations:** ^1^ Department of Nephrology, the First Affiliated Hospital of Chongqing Medical University, Chongqing, China; ^2^ Department of Cardiothoracic Surgery, the First Affiliated Hospital of Chongqing Medical University, Chongqing, China

**Keywords:** type A aortic dissection, hemodialysis, heparin, access patency, case report

## Abstract

**Introduction:**

Type A aortic dissection (AD) is a lethal situation with high mortality within short time after onset. We present here a rare hemodialysis patient whose condition was comorbid with unrepaired type A AD. The challenge we face is whether low-molecular-weight heparin (LMWH) should be used during dialysis.

**Case presentation:**

A 72-year-old man with a history of hemodialysis for 2 years and 7 months sought medical attention due to thrombosis of the dialysis catheter. He had been diagnosed with an unrepaired type A aortic dissection (involving the aortic root, the ascending aorta, the aortic arch, the descending aorta, the abdominal aorta, the left common iliac artery, and the femoral artery) for more than 5 years. LMWH was not given during the previous dialysis process because of concerns about the rupture of the dissection. The lesion was salvaged via urokinase thrombolysis. However, the anticoagulant-free dialysis pattern occasionally caused dialyzer clotting and further increased the risk of catheter dysfunction. The patient repeatedly experienced dysfunction of the catheter in the following 8 months, with 2 episodes resolved via thrombolysis and 2 episodes replaced with new catheters. Finally, LMWH was used for each dialysis session to prevent thrombosis, with the dosage gradually increasing from 1,000 units to 2,000 units. The dosage of 2,000 units could support sufficient 4-hour dialysis for each session. Twenty-five months have passed since then, the patient has not experienced any further occlusion of the catheter, and the aortic dissection has not shown obvious changes (neither obvious expansion nor rupture).

**Conclusion:**

Reducing the dosage of LMWH during hemodialysis is a feasible solution to maintain a balance between hemodialysis access patency and stable dissection status in this particular patient.

## Introduction

Type A aortic dissection (AD) is a lethal situation with high mortality within short time after onset. Although the 30-day or in-hospital mortality of type A AD after surgical repair has decreased to less than 20%, only a scarce group of patients enter the chronic phase without intervention (1–4). With the widespread use of frozen elephant trunk repair technique, the in-hospital mortality rate of type A AD has decreased to below 10% in recent years (5). We present here a rare hemodialysis patient whose condition was comorbid with unrepaired type A AD. As a procedure involving extracorporeal circulation, anticoagulant use is a standard practice during hemodialysis. However, anticoagulants may disrupt the stability of the dissection. There is a lack of literature on how to make decisions in this situation. The challenge we face is whether anticoagulants should be used during dialysis.

## Case report

A 72-year-old man was admitted on 20 April 2022 due to the first episode of dialysis catheter dysfunction. The patient was diagnosed with stage 3 chronic kidney disease in 2012 and developed stage 5 disease in 2019. Because the patient had reached the indications for hemodialysis and the vascular conditions in the upper limbs were too poor to create an arteriovenous fistula or graft, a long-term central venous catheter was placed in the right internal jugular vein in September 2019. Afterwards, the patient underwent regular hemodialysis (3 times per week).

**Figure 1:**
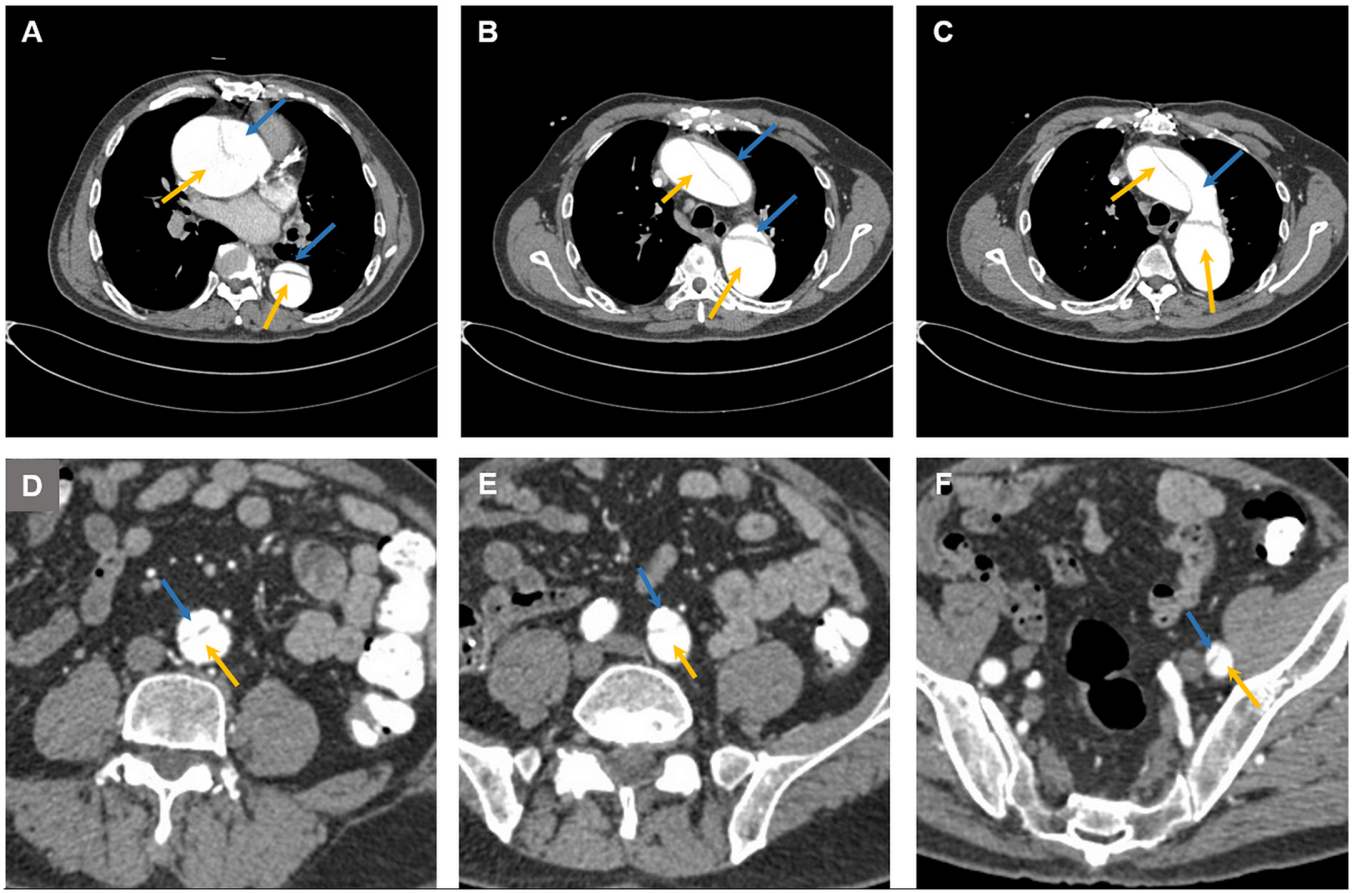
Computed tomography images showing aortic dissection at **(A)** the aortic root, **(B)** the ascending aorta, the descending aorta, **(C)** the aortic arch, **(D)** the abdominal aorta, **(E)** the left common iliac artery, and **(F)** the femoral artery. The blue arrow indicates the false lumen, and the yellow arrow indicates the true lumen.

**Figure 2:**
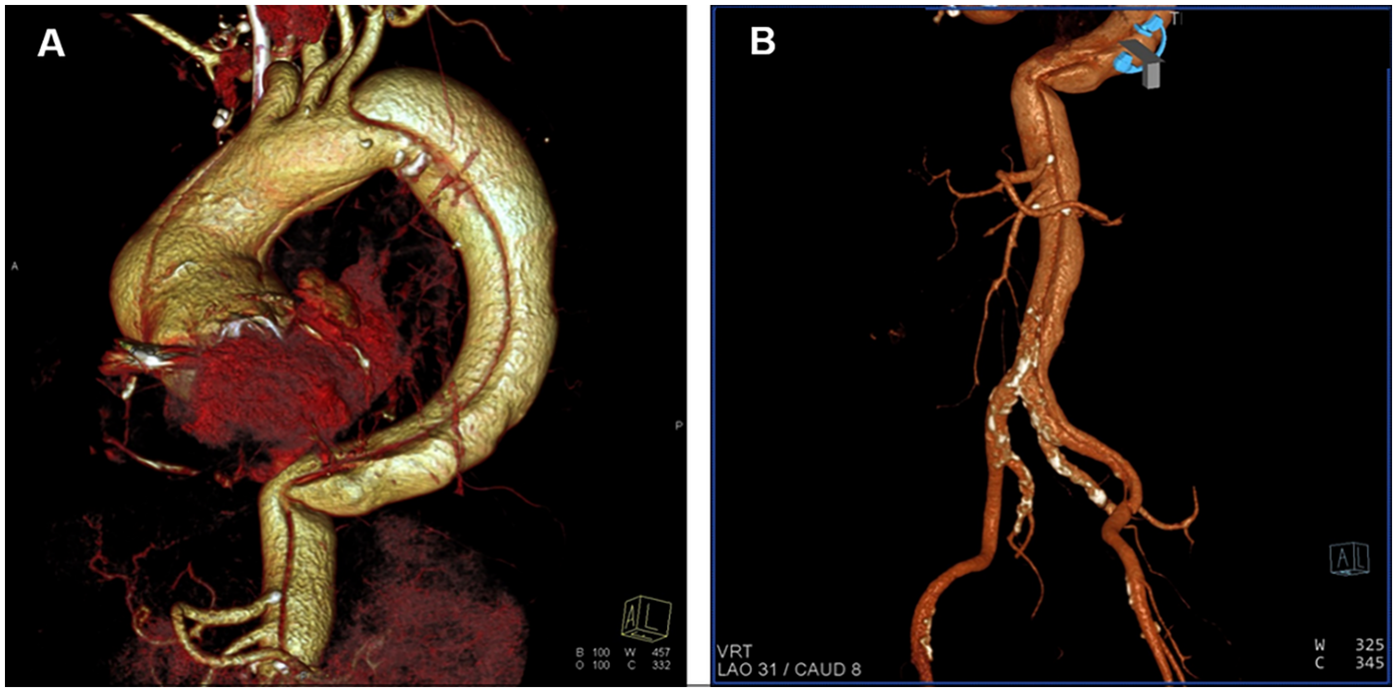
Three-dimensional (3D) reconstruction images from computed tomography angiography. **(A)** Thoracic reconstruction showing dissection of the ascending aorta, the aortic arch, and the descending aorta. **(B)** Abdominal reconstruction showing dissection of the abdominal aorta, the left common iliac artery and the femoral artery.

The patient had a long and complex history of illness (Table 1). The patient had been diagnosed with type A aortic dissection for more than 5 years. Computed tomography (CT) revealed extensive dissection involving the aortic root, the ascending aorta, the aortic arch, the descending aorta, the abdominal aorta, the left common iliac artery, and the femoral artery (Figures 1, 2). The diameter of the ascending aorta remained at 83–89 mm after 2019. Because CT angiography failed to detect the site of the intimal tear and because of the lack of technical capabilities, the patient had not received any repair surgery. When the patient started hemodialysis therapy, low-molecular-weight heparin (LMWH) was not administered because of the concerns about inducing dissection rupture. The anticoagulant-free dialysis pattern frequently caused dialyzer clotting, but could still maintain the patient's normal physiological state.

**Table 1:** Medical history of the patient.

Year	Medical situation	Treatment
2002	The patient was diagnosed with hypertension with a reading of 160/94 mmHg.	Valsartan was administered.
2006	Fasting blood glucose level rose to 7.8 mmol/L.	Not treated.
2007	Discovery of aortic insufficiency during physical examination.	The patient was not treated because there were no obvious symptoms.
2012	The patient was diagnosed with stage 3 chronic kidney disease with a serum creatinine level of 144 μmol/L.	1. Insulin therapy for diabetes started in April and fasting blood glucose was controlled at 6–7 mmol/L. 2. Traditional Chinese medicine was administered for the treatment of chronic kidney disease.
2016	The patient was diagnosed with rheumatic heart disease, aortic valve stenosis with aortic insufficiency, dilatation of the ascending aorta, and coronary atherosclerotic heart disease. The main symptoms included mental fatigue and shortness of breath after the activity, blackouts, chest pain, dizziness, paleness, and dyspnea.	Ascending aortic replacement and aortic valve replacement were performed on April 11th. Postoperative ultrasound revealed normal valve opening and closing with good circulation. The patient was discharged on April 21st.
2017	Postoperative follow-up examination revealed a type A aortic dissection on January 19th. The blood pressure had been controlled at 100–130/70–80 mmHg in the last five years.	No repair surgery was performed because computed tomography did not reveal any intimal tears, and the visiting hospital lacked technical capabilities because of the extensive lesion range.
2019	The obvious symptoms of uremia appeared: glomerular filtration rate <15 ml/min, serum creatinine level >400 μmol/L, edema of both lower limbs, fatigue, shortness of breath after activity, and poor appetite.	A long-term central venous catheter was placed in the right neck in September. Afterwards, the patient underwent regular hemodialysis (3 times per week). Moreover, antihypertensive medication was discontinued due to dialysis-induced hypotension and decrease in blood pressure during non-dialysis period. Systolic blood pressure was maintained at 100–100 mmHg.
2022	The patient experienced repeated fainting and was diagnosed with bradycardia in January.	Implantation of a pacemaker for the treatment of bradycardia.

When dialysis catheter dysfunction occurs, thrombosis (usually located at the opening of the catheter) is the first possible cause. The routine procedure involves first performing urokinase thrombolysis and then aspirating with a syringe. If catheter dysfunction is solely caused by a thrombus, this operation can extract the thrombus and restore blood flow. If urokinase thrombolysis does not work, a fibrin sheath is likely to have formed, and the catheter must be replaced. This episode of catheter dysfunction was salvaged via urokinase thrombolysis, and the patient was discharged. However, the patient experienced an additional 4 episodes of catheter dysfunction in the following 8 months. Two episodes were solved with urokinase thrombolysis (August and October 2022), and the other two episodes were treated with catheter replacement (May and November 2022). The patient was bothered by repeated catheter dysfunction and requested heparin usage during hemodialysis. Given that there is currently no reference on the use of LMWH in this situation, doctors have decided to start with low-dose LMWH in combination with surveillance imaging of the aortic dissection. Typically, 4,000 units of LMWH are administered per hemodialysis session. Starting in January 2023, 1,000 units of LMWH were administered intravenously during each hemodialysis session. The problem of dialyzer clotting improved but still occurred occasionally. The dosage gradually increased to 1,500 units in June 2023 and 2,000 units in July 2023, which enabled the patient to complete 4 h of adequate hemodialysis almost each session. The last follow-up was in December 2024. The patient did not experience new catheter dysfunction after the catheter was changed in November 2022, and the aortic dissection did not significantly change (neither obvious expansion nor rupture).

## Discussion

**Figure 3:**
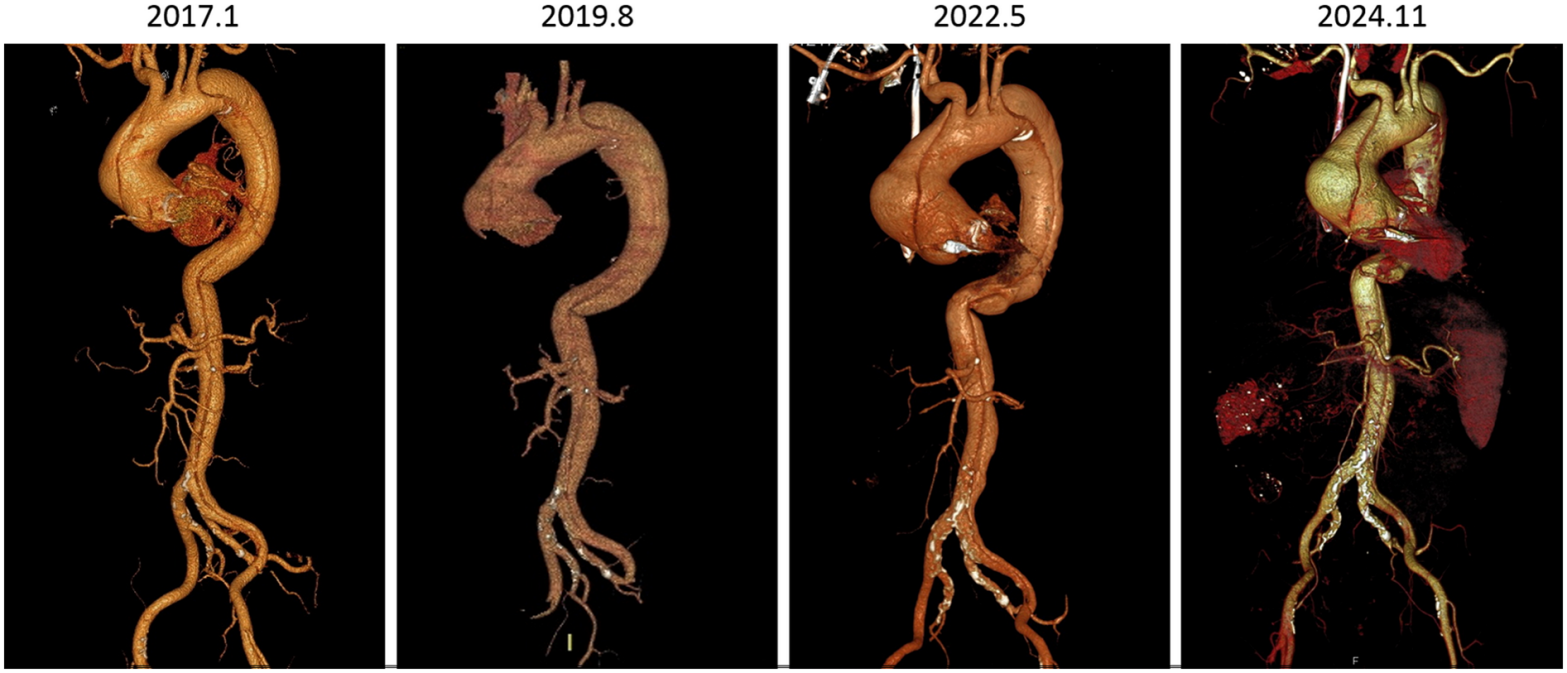
Computed tomography angiography images of different time points.

One notable feature of this case is that the patient has survived stably for over 7 years with unrepaired extensive type A aortic dissection. WK Kim et al. reported that in patients with unrepaired type A AD, the cumulative adverse aortic event rate in those with an aortic diameter ≥55 mm was 27.8% ± 9.1% (6). The diameter of the ascending aorta in this case was 83–89 mmm. The length of stable survival in this case is quite spectacular considering the patient's lesion range, age, aortic diameter, and complex comorbidities. DM Nemtut et al. reported a patient who experienced ascending aortic dissection, descending aortic dissection, and abdominal aortic dissection within a couple of years and eventually died of periaortic hematoma and severe anemia after surgical treatment (7). We have several speculations about the possible mechanism of the patient's long-term survival. First, the patient's blood pressure was well controlled. Second, spontaneous thrombosis probably occurred and blocked the arterial tear because the dissection has not obviously changed since 2019 (Figure 3). Additionally, the aortic dissection may worsen renal function because the renal artery was also affected and dilated, although the direct etiology of renal failure is highly likely to be chronic nephritis.

Uncontrolled hypertension is the most common risk factor for aortic dissection (8). However, the patient's blood pressure had been within the normal range for the last five years before dissection was found. The cause of dissection is highly suspected to be related to the valve replacement surgery. CT angiography did not detect any intimal tear. It is suspected that blood flowed into the intima and media from the site of valve replacement and gradually tore to the femoral artery under gravity. However, this suspicion cannot be confirmed. The CT results of the patient were normal at discharge (10 days post-surgery), and the patient did not experience any severe chest pain before the discovery of the dissection (7 months later). It is even impossible to determine the onset of dissection. The patient's stable state also benefits from controlled blood pressure. After the discovery of the aortic dissection, blood pressure control was strengthened. After hemodialysis was initiated, dehydration during the dialysis process had a synergistic effect on lowering blood pressure. As a result, the patient not only experienced dialysis-related hypotension but also gradually recovered to normal blood pressure during the non-dialysis period and ceased antihypertensive medication. The prevalence of chronic dialysis hypotension was reported to be 8% in long-term dialysis patients (9). However, a literature search did not reveal similar cases of spontaneous recovery of blood pressure in hemodialysis patients. We have no clue about the underlying mechanism.

Naturally occurring thrombus formation in the false lumen may halt the disease progression in AD, but the impact of anticoagulants on the status of AD is unclear (10, 11). On the basis of conservative considerations, LMWH was not used in the current case when initiating hemodialysis. However, the anticoagulant-free dialysis pattern results in frequent dialyzer clotting and further leads to dialysis inadequacy (12). Frequent catheter blockages in our patient were likely also related to the heparin-free dialysis pattern. Small blood clots may flow back into the patient's body, and unfortunately, the catheter is more prone to thrombus deposition than autogenous blood vessels are (13). Thrombosis inside catheters further increases the formation of fibrin sheaths, which is a major cause of catheter malfunction (14, 15). Adopting a compromise heparin dosage (half of the normal dosage) seems to be a relatively appropriate solution, as it can solve the problem of repeated catheter occlusion. Regarding the potential benefits of heparin-free dialysis, a study of 12 thousand patients in the United States revealed that it was associated with neither decreased risk of mortality or bleeding nor increased risk of atherothrombosis or venous thromboembolism (16). Therefore, current attention to the dissection state is focused on maintaining reasonable blood pressure and surveillance imaging, and heparin use does not seem to be a particularly high-risk factor.

In summary, reducing the dosage of heparin during hemodialysis is a feasible solution for balancing aortic dissection status and dialysis patency in this particular patient. Since no similar case has been reported before, our experience can provide a reference for colleagues.

## Data Availability

The original contributions presented in the study are included in the article/Supplementary Material, further inquiries can be directed to the corresponding author.
